# The Food of the People

**Published:** 1903-11-14

**Authors:** 


### THE FOOD OF THE PEOPLE.

We have more than once expressed the opinion that members of the medical profession, especially those living and practising in rural districts, might not only obtain and exert a greater influence than they now possess over the habits of life of the people, but that also, in striving to obtain and to employ this influence, they might win for the profession itself a better position in the public estimation than it now actually holds, and might justify its claim to be a guide in many matters which bear a real relation to health, even when this relation is not immediately apparent upon the surface. Among these, few can be of greater importance than the national dietary. In an article on Feeding and Disease, last week, we referred casually to Mrs. Scatherd's mention, at the Bradford Conference, of the "deleterious effects of patent foods," and it is manifest that the very suggestion of such an influence is in the highest degree worthy of medical consideration. Mr. Chamberlain is telling great audiences that, in the ordinary course of nature and of affairs, we must look forward, perhaps at no distant time, to a period of depression of trade, during which the aggregate earnings of our industrial classes may be greatly diminished, and the problem of how to make both ends meet may become a very pressing one to thousands of families. Under such conditions, the judicious purchase of food would become a matter of primary importance, not only as regards the preservation of the strength of the bread-winner while he was waiting for better times, but also as regards the ultimate physical capacities of his growing children. It is obvious that scanty means may be laid out either wisely or foolishly, and that, when large numbers of people are concerned, the national welfare over a long period may be seriously affected by the result. Nothing can be more important, even in times of prosperity, than that our working classes should obtain good pennyworths for their pennies, or that the wives and mothers among them should be able to cater judiciously for the wants of their families. We may perhaps hope for the coming of a time when careful instruction in such matters will hold its proper place in the system of national education.

In the meanwhile, the ordinary housewife among the artisan class has no knowledge by which she can be safely guided, and no accessible referee by whom she can be judiciously advised. She is frequently unskilled in even the simplest forms of cookery, and she sees the walls in her neighbourhood covered by flaming posters, with more or less skilful and attractive illustrations, and with letterpress setting forth the advantages to be gained by the purchase of this or that preparation of meat, of vegetables, or of cereals. Most of these preparations have the common character of being easy to prepare for the table, and of being palatable when the simple directions sold with them have been followed, but the question of their nutritive value is not only a totally different one, but is often one of considerable difficulty, on which skilled guidance is imperatively required. Gelatine was believed to be highly nutritious, until Majendie showed that dogs fed upon it died of starvation as quickly as dogs that were not fed at all; but even now a belief in its value has not been entirely dissipated, and the setting of soup into a jelly is regarded by many as an evidence of its goodness for dietetic purposes. If any of the largely advertised patent foods do not really possess the nutritive value that is claimed for them in posters and advertisements, the purchasers are to that extent defrauded, and it is at least conceivable that the consequences of the fraud may be serious and permanent. Again to quote Mr. Chamberlain, it is the part of wisdom to adapt our policy to changed or changing conditions; and, at a time when strenuous endeavours are being made by advertisers to induce the purchase of new articles of food, it would be sound policy to commit to a Government department the task of investigating their actual value, as measured by the familiar standards of bread and meat. The poor want to know, on trustworthy authority, whether it is really cheaper and better to buy a packet of patent food than to buy a quartern loaf; and there is no one who can tell them. If the claims advanced on behalf of the packet have any foundation in fact, the sellers of it should be the last people to object to a proper estimation of their wares; but it should be remembered that many of the preparations referred to are of American origin, and that the conscience of the American advertiser is commonly believed to be at least as elastic as the corresponding article among nonconformists. Pending a better way of bringing about the same result, we think that a country doctor might without much difficulty attain to a sufficient knowledge of the real merits or demerits of the patent foods most freely advertised in his locality, and that he might say a word in season to the clergyman and to the farmer's wife which would in due course penetrate to the cottage.

